# Chronotropic Incompetence in Parkinson’s Disease: A Possible Marker of Severe Disease Phenotype?

**DOI:** 10.3233/JPD-230256

**Published:** 2024-04-23

**Authors:** Mattias Andréasson, Jannike Nickander, Marcus Ståhlberg, Artur Fedorowski, Per Svenningsson

**Affiliations:** a Center for Neurology, Academic Specialist Center, Stockholm, Sweden; b Department of Neurology, Karolinska University Hospital, Stockholm, Sweden; c Department of Clinical Neuroscience, Karolinska Institutet, Stockholm, Sweden; d Department of Clinical Physiology, Karolinska University Hospital, and Karolinska Institutet, Stockholm, Sweden; e Department of Cardiology, Karolinska University Hospital, Solna, Stockholm, Sweden; f Department of Medicine, Karolinska Institutet, Solna, Stockholm, Sweden

**Keywords:** Parkinson’s disease, autonomic nervous system diseases, postganglionic autonomic fibers, exercise test

## Abstract

Autonomic dysfunction is a prevalent feature of Parkinson’s disease (PD), mediated by disease involvement of the autonomic nervous system. Chronotropic incompetence (CI) refers to inadequate increase of heart rate in response to elevated metabolic demand, partly dependent on postganglionic sympathetic tone. In a retrospective study, PD patients with/without CI were identified. We show that PD with CI was associated with a higher levodopa equivalent daily dose and Hoehn and Yahr stage, 5±2 years after motor onset. Our data support a putative role of CI as a clinical marker of a more severe disease phenotype, possibly reflecting more widespread alpha-synuclein pathology.

## INTRODUCTION

Autonomic dysfunction is a well-established feature of Parkinson’s disease (PD) and a wide spectrum of autonomic symptoms may present both before and after motor onset [1, 2]. Pathology involving the autonomic nervous system has been suggested to contribute to this symptomatology [3].

Chronotropic incompetence (CI) reflects the inability to adequately increase heart rate in response to elevated metabolic demand and may reflect cardiac autonomic dysfunction of varying underlying etiology. Apart from cardiac diseases affecting sinus node automaticity and cardiac electric signal conduction (e.g., sick sinus syndrome, sinoatrial dysfunction, and atrioventricular conduction disorders), disturbed cardiovagal regulation and disruption of postganglionic sympathetic cardiac innervation are possible underlying causes [4, 5]. A cardiac stress test is a method to assess the presence of CI under controlled conditions in the laboratory, and has been proposed to be defined as the inability to achieve > 85% of age-expected maximum heart rate during testing [6, 7].

Postganglionic sympathetic cardiac denervation has repeatedly been demonstrated in PD through neuroimaging [8] and neuropathological studies [9]. We therefore performed a retrospective analysis of patients with PD who had a record of undergoing a cardiac stress test, with the aim of comparing clinical features stratified by the presence of CI.

## MATERIALS AND METHODS

In a retrospective manner, data was extracted from the ongoing observational BioPark Study that, since 2011, includes patients with parkinsonism at Karolinska University Hospital and Center for Neurology, Stockholm, Sweden. All participants have given written informed consent (ethical approval ref.nr 2011_500-31_1; 2016/19-31/1; 2019-04967) and in accordance with the Helsinki declaration.

**Fig. 1:**
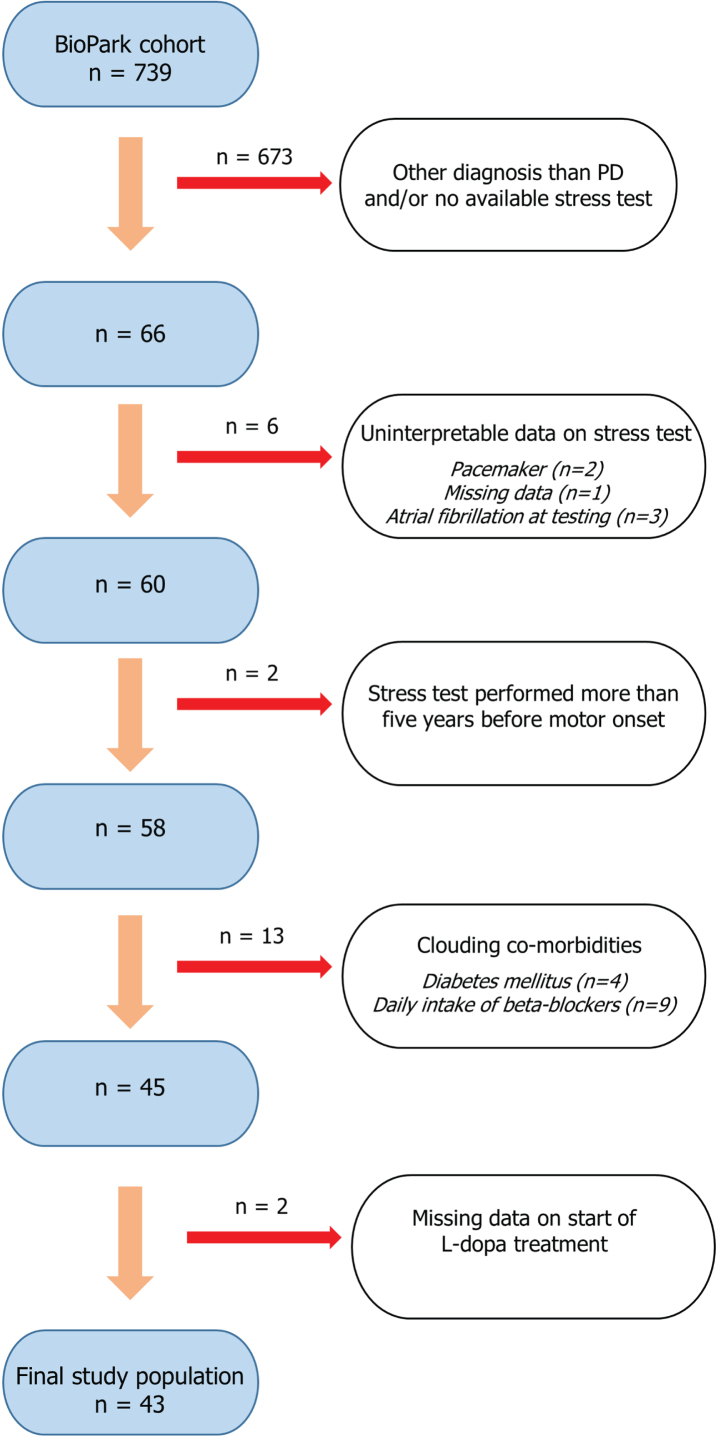
Flowchart illustrating the stepwise process of selecting the study population (*n* = 43). Patients with parkinsonism of different neurodegenerative etiologies are prospectively included in the ongoing observational BioPark Study. Stepwise selection of the current study population included exclusion of patients not meeting criteria for Parkinson’s disease, no available record of previous cardiac stress test, missing/uninterpretable data and if the stress test was performed more than five years before motor onset. Furthermore, patients with co-morbid conditions possibly associated with chronotropic incompetence were excluded.

At the time of data extraction, December 2022, 739 participants were identified. A flowchart illustrating the selection process for the current study population is shown in Fig. 1. Shortly, by reviewing medical records (*n* = 739), 66 patients with PD and a previously recorded cardiac stress test were identified. Participants with no record on start of levodopa (L-dopa) treatment, and co-morbidities known to impact a cardiac stress test (daily treatment with beta-blockers, pacemaker, atrial fibrillation, and diabetes mellitus) were excluded. Furthermore, given the uncertainty of when the neurodegenerative disease process starts, stress tests performed more than five years before motor onset were excluded (*n* = 2). This time criterion is supported by a previous retrospective study that suggested CI may present as early as four years before motor onset in PD [10]. In patients with multiple tests recorded, the most recent evaluation was used. Accordingly, the timing of the stress test differed between patients and was not performed at a standardized time point relative to the date of motor onset. A final study population of 43 patients was included for further analysis.

The study population was dichotomized with regard to the presence of CI, defined as:

### Chronotropic incompetence: achieved maximum heart rate/(220-age) <0.85 [6]

Additional physiological variables were extracted from stress test reports. These included resting heart rate prior to start of the test, and the presence of a blunted blood pressure (BP) response, defined as an increase of less than 50 mmHg or a drop of more than 20 mmHg, during the test. The achieved maximum workload during the test was calculated as the percentage of the expected maximum workload, based on a Swedish reference material [11]. Available clinical and demographic data were extracted, including the modified Hoehn and Yahr (mHY) stage [12, 13] and the L-dopa equivalent daily dose (LEDD) [14] at a fixed time point 5±2 years after motor onset.

Non-parametric statistics were used given the non-normal distribution of variables. Median and interquartile range are presented for numerical and ordinal variables, and proportions for categorical variables. Mann-Whitney U-test and Fisher’s Exact test were used to compare numerical and categorical variables between groups, respectively. Correlation analyses were performed with Spearman’s rank order correlation test. A *p*-value <0.05 was considered significant.

## RESULTS

Clinical characteristics of the study population (*n* = 43) are shown in Table 1. Patients with (PD + CI) and without CI (PD-CI) were comparable with regard to sex, age at motor onset, and age at time of cardiac stress test. PD + CI achieved a significantly lower maximum workload compared to PD-CI (*p* = 0.0065). A blunted BP response was more common in PD + CI (*p* = 0.015).

**Table 1:** Study cohort (*n* = 43)

	PD + CI (*n* = 18)	PD-CI (*n* = 25)	*p*
Age at motor onset (y), median (IQR)	62.7 (17.3)	61.7 (12.2)	0.52
Sex (M/F)	13/5	17/8	1.0
Smoking (*n*, % yes)^*^	1 (5.6)	0 (0)	0.43
Cardiovascular co-morbidities
Hypertension (*n*, % yes)	9 (50)	7 (28)	0.20
Renal disease (*n*, % yes)	0 (0)	1 (4.0)	1.0
Ischemic heart disease (*n*, % yes)	2 (11.1)	0 (0)	0.17
Congestive heart failure (*n*, % yes)	1 (5.6)	0 (0)	0.42
Prior cardiac ablation (*n*, % yes)	0 (0)	1 (4.0)	1.0
Cardiac stress test
Age at stress test (y), median (IQR)	69.2 (12.8)	62.1 (16.8)	0.16
Time after motor onset (y), median (IQR)	3.7 (9.0)	3.4 (9.1)	0.23
Stress test prior to motor onset (*n*, % yes)	2 (11.1)	6 (24)	0.43
Achieved max. workload (% of expected), median (IQR)^**^	0.68 (0.27)	0.88 (0.20)	**0.0065**
Resting heart rate (beats/min), median (IQR)^***^	66.5 (9.0)	72.5 (15.8)	0.060
Maximum heart rate (beats/min), median (IQR)	113.5 (14.3)	157.0 (26)	**<0.001**
Maximum heart rate (% of expected), median (IQR)	0.78 (0.089)	0.97 (0.096)	**<0.001**
*Δ*Heart rate (beats/min), median (IQR)^***^	51.5 (15.0)	83.0 (22.3)	**<0.001**
Other findings on cardiac stress test
Blunted BP response (*n*, % yes)	6 (33.3)	1 (4.0)	**0.015**
Hypertensive BP response (*n*, % yes)	0 (0)	1 (4.0)	1.0
Possible signs of coronary ischemia (*n*, % yes)	3 (16.7)	2 (8.0)	0.63

Demographic and clinical characteristics of the study cohort are compared between patients with and without chronotropic incompetence. Chronotropic incompetence is defined as < 85 % of expected maximum heart rate achieved during cardiac stress test. Smoking status and cardiovascular co-morbidities reflect data collected at the time of cardiac stress test. Data related to the cardiac stress test reflect different physiological parameters obtained from the test as follows: Achieved maximum workload: The achieved workload (W) during stress test in relation to the expected workload (%) based on Swedish population studies. Resting heart rate: The heart rate detected by electrocardiogram at rest prior to starting stress test. Maximum heart rate: The maximal heart rate detected by electrocardiogram during the stress test. *Δ*Heart rate: The change in heart rate from rest to stress during the stress test. Blunted BP response: A blood pressure increase of less than 50 mmHg, or a drop in blood pressure of more than 20 mmHg during stress test. Hypertensive BP response: A systolic blood pressure of more than 240 mmHg during stress test, according to local clinical routine. Possible signs of coronary ischemia: Include ST-depression and/or ST-elevation, angina, and ventricular arrhythmias during stress test. Data are presented with non-parametric descriptive statistics; median and interquartile range for numerical variables, and proportions (%) for categorical variables. Comparisons were performed with Fisher’s Exact test for categorical and Mann-Whitney U-Test for numerical variables. PD + CI, Parkinson’s disease with chronotropic incompetence; PD-CI, Parkinson’s disease without chronotropic incompetence; y, years; IQR, interquartile range; BP, blood pressure ^*^1 missing value, calculations performed on valid cases (PD + CI, *n* = 18; PD-CI, *n* = 24). ^**^3 missing values, calculations performed on valid cases (PD + CI, *n* = 18; PD-CI, *n* = 22). ^***^7 missing values, calculations performed on valid cases (PD + CI, *n* = 16; PD-CI, *n* = 20).

LEDD and mHY stage 5±2 years after motor onset were significantly higher in PD + CI (*p* = 0.031 and *p* = 0.0052, respectively) (Table 2). Median time intervals between motor onset and the recording of LEDD and mHY were comparable between groups (PD + CI: 5.15 vs. PD-CI: 5.11 years, *p* = 0.87; PD + CI: 5.21 vs. PD-CI: 5.09 years, *p* = 0.80, respectively). Studying the whole population, a negative association was demonstrated between the achieved percentage of expected maximum heart rate (% max HR) and mHY stage 5±2 years after motor onset (rho = –0.51, *p* = 0.0011), and reached borderline significance in relation to LEDD (rho = –0.29, *p* = 0.058). Time from motor onset to start of L-dopa treatment did not differ significantly between groups (*p* = 0.75), nor was any association seen with % max HR.

**Table 2:** Group comparisons of possible markers of disease burden

	PD + CI (*n* = 18)	PD-CI (*n* = 25)	*p*
LEDD^*^ (mg), median (IQR)	598 (369)	400 (370)	**0.031**
mHY stage^**^, median (IQR)	2.0 (0.5)	2.0 (1.0)	**0.0052**
Distribution^**^			**0.033**
1.0 (*n*, %)	2 (12.5)	6 (27.3)
1.5 (*n*, %)	0 (0)	4 (18.2)
2.0 (*n*, %)	9 (56.3)	12 (54.5)
2.5 (*n*, %)	3 (18.8)	0 (0)
3.0 (*n*, %)	1 (6.3)	0 (0)
4.0 (*n*, %)	1 (6.3)	0 (0)
Time to L-dopa (y), median (IQR)	2.7 (2.2)	2.8 (4.8)	0.75

The L-dopa equivalent daily dose and modified Hoehn and Yahr stage recorded 5±2 years after motor onset are shown. Groupwise comparisons demonstrate significant differences suggestive of a higher disease burden in patients with chronotropic incompetence. Data are presented with non-parametric descriptive statistics; median and interquartile range for numerical and ordinal variables, and proportions (%) for categorical variables. Comparisons were performed with non-parametric Mann-Whitney U-Test, and with regard to mHY stage also with Fisher-Freeman-Halton Exact test. PD + CI, Parkinson’s disease with chronotropic incompetence; PD-CI, Parkinson’s disease without chronotropic incompetence; LEDD, L-dopa equivalent daily dose; IQR, interquartile range; mHY, modified Hoehn and Yahr stage; Time to L-dopa, time from motor onset to start of L-dopa treatment. ^*^1 missing value, calculations performed on valid cases (PD + CI, *n* = 17; PD-CI, *n* = 25). ^**^5 missing values, calculations performed on valid cases (PD + CI, *n* = 16; PD-CI, *n* = 22).

The inclusion of patients with a normal stress test before motor onset may constitute a risk of selection bias. Therefore, in a sensitivity analysis these patients (*n* = 6) were excluded. The main study results remained significant, with a higher LEDD and mHY 5±2 years after motor onset (*p* = 0.029 and *p* = 0.0080, respectively), and an association between % max HR and mHY (rho = –0.49, *p* = 0.0042).

## DISCUSSION

In this study, we retrospectively investigated a cohort of PD patients who had undergone a cardiac stress test. Using a fixed time point, 5±2 years after motor onset, we compared possible features of disease severity stratified by the presence of CI. We found that patients with CI exhibited a significantly higher LEDD and mHY stage compared to patients without CI (Table 2). Furthermore, we explored possible associations between % max HR and clinical outcomes and demonstrated a significant association with mHY.

Autonomic dysfunction [15, 16], including orthostatic hypotension, as well as peripheral large fiber neuropathy [17], have previously been suggested as markers of a more severe disease phenotype in PD with regard to survival, motor and cognitive functions. Moreover, a high autonomic symptom burden at PD symptom onset has been associated with a faster disease progression [18]. Given the results from the current study, we speculate whether CI may serve as an additional marker of severe disease phenotype in PD, as reflected by higher LEDD and mHY stage five years after motor onset.

Numerous studies have reported the detection of pathological phosphorylated alpha-synuclein in peripheral autonomic nerve fibers [19–21]. Furthermore, a previous retrospective study followed patients that had undergone a cardiac stress test for the subsequent development of PD, and suggested that CI indeed may be a premotor feature [10]. Moreover, in a prospective cohort study of patients with pure autonomic failure, a reduced increase in heart rate during head-up tilt test was associated with future phenoconversion to PD but not multiple system atrophy [22]. A putative predictive and diagnostic role of cardiovascular autonomic dysfunction, including CI, has thus been suggested [22, 23].

We hypothesize that CI may be a putative marker of more widespread alpha-synuclein pathology in PD, extending beyond the central nervous system, and as a result contribute to a more severe disease phenotype. However, this hypothesis must be further tested in a prospective manner, possibly by combining cardiac stress test with additional neuropathological and functional assessments of postganglionic sympathetic fibers by means of skin biopsy and quantitative sudomotor axon reflex testing, respectively.

A blunted BP response was seen more commonly in PD + CI. When reviewing these patients (*n* = 6), five had undergone an echocardiogram at least one year before, or at a later time point, without evidence of hemodynamically significant heart disease, and none had evidence of coronary artery disease, the most common causes of blunted BP response [24]. However, one patient had evidence of tricuspid insufficiency grade 2.5/4. The sixth patient had no available echocardiogram, but the electrocardiogram was normal at the time of stress test. Postganglionic sympathetic innervation of blood vessels is, together with cardiac sympathetic innervation, essential for BP regulation by means of determining cardiac output and total peripheral resistance. We speculate that PD + CI may be associated with extracardiac postganglionic sympathetic failure, in line with previous evidence of extracardiac noradrenergic denervation in PD with orthostatic hypotension [25], and thus partly explain our finding of an increased prevalence of blunted BP response in PD + CI. Moreover, cardiovascular regulation during exercise is also dependent on afferent nerve signaling from skeletal muscle to the autonomic nervous system - the exercise pressor reflex. A subset of these muscle afferents, sensitive to metabolic changes within the muscle, has been suggested to be selectively impaired in PD and contribute to a blunted BP response during exercise [26]. Thus, further studies addressing a possible association between PD + CI and impaired muscle metaboreflex may be warranted.

The current study is exploratory in its nature and several limitations need to be mentioned. Cardiac evaluation was not performed at a uniform time point relative to the time of motor onset and the five-year time frame used for the recording of study outcomes (LEDD and mHY). Given that PD is a progressive disorder, it is possible that several CI negative patients developed CI at a later time point. The presented correlation analyses should thus be interpreted with caution.

The small sample size constitutes an additional limitation of the study. Given the exploratory nature of the study, we believe our results still may be of interest and serve as an impetus for future well-powered studies addressing the possible role of PD + CI as a disease subtype with prognostic properties. The study had limited clinical outcome data recorded, including some missing values as reported in Tables 1 and 2, partly owing to the fixed time point at 5±2 years after motor onset. Further assessments on motor and non-motor symptoms would have shed further light on the putative prognostic role of CI in PD.

Furthermore, we believe that autonomic dysfunction may have contributed to the lower achieved maximum workload in PD + CI, although reverse causation cannot be excluded, i.e., more severe parkinsonism may have limited the maximum workload.

Lastly, although cardiovascular co-morbidities were not significantly different between groups at the time of cardiac stress test (Table 1), we cannot exclude the subsequent development of additional cardiovascular diseases in the PD + CI group, which partly may have contributed to the findings of CI.

In conclusion, the presence of CI may be associated with a more severe disease phenotype in PD. We propose that CI may be a clinical marker of postganglionic cardiac denervation associated with peripheral PD pathology, akin to the recently proposed “body-first” PD subtype [27]. Our results merit further evaluation in prospective study designs performed in early disease, with the aim of exploring the possible existence of a PD subtype characterized by CI or a more general peripheral cardiovascular autonomic denervation.

## Data Availability

The data supporting the findings are available on request from the corresponding author.
